# Neuropeptides and Behaviors: How Small Peptides Regulate Nervous System Function and Behavioral Outputs

**DOI:** 10.3389/fnmol.2021.786471

**Published:** 2021-12-02

**Authors:** Umer Saleem Bhat, Navneet Shahi, Siju Surendran, Kavita Babu

**Affiliations:** ^1^Centre for Neuroscience, Indian Institute of Science, Bengaluru, India; ^2^Department of Biological Sciences, Indian Institute of Science Education and Research, Mohali, India

**Keywords:** neuropeptides, *C. elegans*, locomotion, behavior, signaling

## Abstract

One of the reasons that most multicellular animals survive and thrive is because of the adaptable and plastic nature of their nervous systems. For an organism to survive, it is essential for the animal to respond and adapt to environmental changes. This is achieved by sensing external cues and translating them into behaviors through changes in synaptic activity. The nervous system plays a crucial role in constantly evaluating environmental cues and allowing for behavioral plasticity in the organism. Multiple neurotransmitters and neuropeptides have been implicated as key players for integrating sensory information to produce the desired output. Because of its simple nervous system and well-established neuronal connectome, *C. elegans* acts as an excellent model to understand the mechanisms underlying behavioral plasticity. Here, we critically review how neuropeptides modulate a wide range of behaviors by allowing for changes in neuronal and synaptic signaling. This review will have a specific focus on feeding, mating, sleep, addiction, learning and locomotory behaviors in *C. elegans*. With a view to understand evolutionary relationships, we explore the functions and associated pathophysiology of *C. elegans* neuropeptides that are conserved across different phyla. Further, we discuss the mechanisms of neuropeptidergic signaling and how these signals are regulated in different behaviors. Finally, we attempt to provide insight into developing potential therapeutics for neuropeptide-related disorders.

## Introduction

Change is constant! Evolutionary studies show that organisms evolve by adapting to ever-changing environmental conditions. It is therefore critical for an animal’s survival to detect a diverse array of cues. This unique phenomenon of adaptation is attributed to synaptic plasticity [reviewed in Fusco and Minelli (2010)]. Consistent encounter with a stimulus reinforces neuronal wiring to ensure the appropriate biological activity, manifested as behavior [reviewed in Citri and Malenka (2008)]. Physiological activities coupled with biological events during behavior result from the interplay between the brain and the surroundings of an organism. The coordinated action of the neuronal connectome integrates information and directs behavioral responses.

It is intriguing to understand how organisms perceive their environment to execute behaviors and learn from their experiences. Hence, behavioral studies have been of keen interest for researchers in the field of neuroscience. Various reports from the past few decades have made it possible to parse out certain intricacies associated with behaviors and the neuronal and synaptic changes behind these behaviors. These studies have laid the path for delving further to elucidate the mechanisms underlying nervous system processes that direct the required behavioral output. Recent advances in molecular tools have proved a boon for such studies, but several challenges of different magnitudes pose limitations. One of the main challenges is the brain’s complex structure and function with millions of neurons and synaptic connections as seen in most organisms with complex behavioral outputs. To overcome this challenge, *C. elegans* has proved to be a pioneering organism.

The simple nervous system of a *C. elegans* hermaphrodite has just 302 neurons and has been completely reconstructed with electron microscopy (White et al., 1986). Further, *C. elegans* shows discrete, robust, and easily quantifiable behaviors, making it a suitable model system. Often, these behavioral studies in worms revolve around the wired neuronal network consisting of synaptic connections by small classical neurotransmitters. However, this review will focus on the non-wired neuronal network that involves the transmission of information by neuropeptidergic signaling. Unlike classical neurotransmitters that function through wiring transmission, neuropeptides function through volumetric transmissions and play a critical role in sustained biological responses (Sorensen et al., 2008; van den Pol, 2012). Neuropeptides are also known to modulate the activity of co-released neurotransmitters to increase or decrease the strength of synaptic signaling [reviewed in Russo (2017)]. Noteworthy is that these small peptides can also act as peptidergic hormones to regulate other bodily functions. Therefore, neuropeptides have been established as modulators of behavior in a wide range of animals. In *C. elegans*, neuropeptides are classified into three different families, viz, FMRFamide or FLP-like peptides (FLPs), Insulin-like peptides (ILPs), and Neuropeptide-like proteins (NLPs) [reviewed in Holden-Dye and Walker (2013)]. Evidence that these diverse neuropeptides play important roles in locomotion, mating, learning and memory, sleep and addiction is accumulating, but an integration has been lacking. Even though the functions of neuropeptides in *C. elegans* have been vigorously studied, relatively little is known about their modes of action in modulating behavior. Here, we attempt at piecing together the available information, to construct mechanistic models of behaviors regulated by neuropeptides. The list of all neuropeptides found in *C. elegans* that are discussed in this review can be found in Table 1.

**TABLE 1:** List of neuropeptides discussed in this review.

**S. no.**	**Neuropeptides**	**Behavioral defects associated with neuropeptide mutants**	**Receptor/s (if known)**	**References**
1.	FLP-1	Bending angle/fat storage/food- evoked foraging	NPR-6/FRPR-7/NPR-4/NPR-9	Nelson et al. (1998), Oranth et al. (2018), Jia and Sieburth (2021)
2.	FLP-2	Arousal	FRPR-18	Chen et al. (2016)
3.	FLP-3	Swimming	-	Chang et al. (2015)
4.	FLP-5	Mating	-	Lints et al. (2004)
5.	FLP-6	Mating	-	Lints et al. (2004)
6.	FLP-7	Feeding/fat mobilization	NPR-22	Palamiuc et al. (2017)
7.	FLP-8	Mating	-	Liu et al. (2007)
8.	FLP-10	Mating/swimming	-	Liu et al. (2007), Chang et al. (2015)
9.	FLP-11	Sleep	-	Turek et al. (2016)
10.	FLP-12	Mating	-	Liu et al. (2007)
11.	FLP-13	Sleep	FRPR-4	Nelson et al. (2014), Nath et al. (2016)
12.	FLP-17	Feeding/mating	-	Lints et al. (2004), Dalliere et al. (2016)
13.	FLP-18	Reversals/swimming/foraging/feeding	NPR-1/NPR-4/NPR-5	Cohen et al. (2009), Chang et al. (2015), Lemieux et al. (2015), Bhardwaj et al. (2018, 2020)
14.	FLP-20	Reversals/arousal/mating/learning and memory	FRPR-3	Liu et al. (2007), Li et al. (2013), Rabinowitch et al. (2016), Chew et al. (2018b)
15.	FLP-21	Social feeding	NPR-1	Rogers et al. (2003), Chang et al. (2015)
16.	FLP-24	Sleep	-	Nath et al. (2016)
17.	FLP-34	Learning and memory	NPR-11	Fadda et al. (2020)
18.	NLP-8	Sleep	-	Nath et al. (2016)
19.	NLP-12	Number and amplitude of body Bends/feeding/fat storage	CKR-2	Janssen et al. (2008), Hu et al. (2011), Bhattacharya et al. (2014), Pandey et al. (2021)
20.	NLP-22	Sleep	-	Nelson et al. (2013)
21.	NLP-24	Feeding	NPR-17	Cheong et al. (2015)
22.	NLP-38	Learning and memory	SPRR-2	Peymen et al. (2019)
23.	NLP-49	Number and angle of body- bends/arousal	SEB-3	Chew et al. (2018a)
24.	PDF-1, PDF-2	Mating/reversals/sleep and lethargus	PDFR-1/PDFR-2	Barrios et al. (2012), Choi et al. (2013), Flavell et al. (2013), Hilbert and Kim (2018)
25.	INS-1	Food adaptation	-	Chalasani et al. (2010), Dwyer and Aamodt (2013)
26.	INS-6	Olfactory Learning	-	Chen et al. (2013)
27.	INS-7	Learning	-	Chen et al. (2013)
28.	INS-11	Learning and memory	-	Lee and Mylonakis (2017)
29.	Luqin-like RYamide peptides	Food evoked satiety	NPR-22	Ohno et al. (2017)
30.	Nematocin (NTC-1)	Mating	NTR-1	Garrison et al. (2012)
31.	RGBA-1	Mating	NPR-28	Yin et al. (2017)
32.	Neuromeric-U (NMU)	Learning and memory	NMUR-1	Watteyne et al. (2020)

### Locomotion

Locomotion is a fundamental life process for all organisms to survive and thrive. It is the basis for numerous behaviors like foraging, feeding, mating, escaping predators, sleep, migration, and dispersal [reviewed in Gjorgjieva et al. (2014)]. The locomotion pattern differs across organisms and involves walking, running, flying, swimming, and crawling, depending upon the organism’s body plan.

**FIGURE 1:**
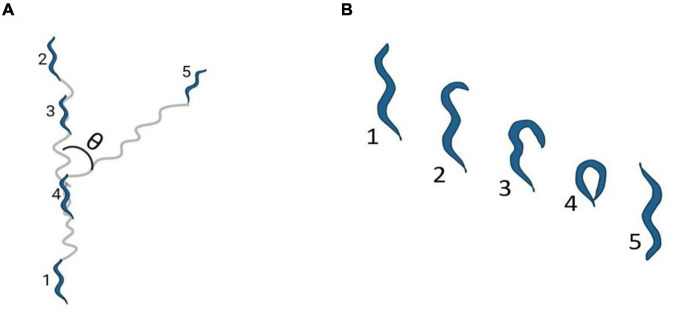
Locomotion Pattern of *C elegans*. **(A)** Shows reversals, where θ represents the bending angle during reorientation and the numbers 1–5 indicate the stages of reversals in order **(B)** indicates an Ω turn where the numbers 1–5 indicates the stages leading to an Ω turn in order. The images have been adapted from Gray et al. (2005).

In *C. elegans*, locomotion is an integral part of nearly all behaviors. *C. elegans* move in a sinusoidal pattern as a result of the dorsoventral flexing of body wall muscles. The differential synaptic inputs on these muscles restricts the movement in these worms to dorsoventral turns. The propagation of sinusoidal waves determines the direction of motion (White et al., 1986). Movement in *C. elegans* is a combination of different motion patterns, including forward crawls, reverse crawl (also known as reversals, illustrated in Figure 1A), and omega (Ω) turns [sharp reorientation events in which the head almost touches the tail, illustrated in Figure 1B and in Gray et al. (2005)]. It is interesting to note here that the frequency of reversals and Ω turns is critical in shaping *C. elegans* trajectory while executing any locomotory behavior (Gray et al., 2005; Lopez-Cruz et al., 2019).

**FIGURE 2:**
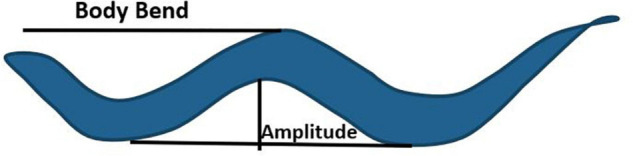
Body bend and amplitude of a sinusoidal wave during locomotion in *C. elegans.* The image has been adapted fromPandey et al. (2021).

Locomotion, albeit a complex behavior controlled by wired and non-wired neuronal circuitry, and is regulated by environmental and internal factors. Although the cues and neuronal connectome controlling locomotion have been largely characterized, the underlying molecular mechanism remain to be fully elucidated (Gray et al., 2005; Piggott et al., 2011). Apart from the conventional small neurotransmitters, neuropeptides also play a critical role in shaping locomotion in response to perturbations in the system. Fundamental studies exploring the role of neuropeptides in locomotion were based on mutations in two genes required for formation of neuropeptides, EGL-3 (Proprotein convertase) and EGL-21 (Carboxypeptidase E). Proteins encoded by these genes are required for the maturation of neuropeptides into their functional forms (Kass et al., 2001; Jacob and Kaplan, 2003). Mutants in *egl-3* and *egl-21* show decreased sensitivity to the acetylcholine esterase inhibitor, aldicarb, as well as decreased acetylcholine (ACh) release in the presence of aldicarb at the neuromuscular junction (NMJ) (Jacob and Kaplan, 2003; Hu et al., 2011). Moreover, mutants in the neuropeptide *nlp-12* show resistance to aldicarb, again suggesting that neuropeptides regulate the levels of acetylcholine at NMJ (Hu et al., 2011). NLP-12 (mammalian homolog of cholecystokinin), released from the DVA neuron, binds to its receptor CKR-2 on cholinergic motor neurons to allow for regulating ACh release at the NMJ, this in turn regulates locomotion in *C. elegans* (Hu et al., 2011). NLP-12 also integrates dopamine and ACh signaling pathways as DVA is postsynaptic to the PDE dopaminergic neuron. This integrated pathway when activated, increases body bends and amplitude of sinusoidal wave during locomotion to promote dwelling while on food (body bends and amplitude are illustrated in Figure 2; Bhattacharya et al., 2014; Bhattacharya and Francis, 2015). In contrast, the absence of food is associated with reduced dopamine levels and dispersal behavior (Sawin et al., 2000). The circuitry for this behavior has been delineated by Oranth et al. (2018). They show that PDE inhibits the AVK interneuron through dopamine/DOP-3 signaling in the presence of food. AVK releases the FLP-1 neuropeptide, which binds to receptors NPR-6 and FRPR-7 on the ventral cord and head motor neurons. FLP-1 inhibits the motor neurons and promotes dispersal by reducing bending angles during locomotion. Previous studies have also shown that FLP-1 maintains the amplitude of sinusoidal waves (Nelson et al., 1998), rate of body bends during swimming (Chang et al., 2015; Buntschuh et al., 2018), and the excitation-inhibition balance during locomotion (Stawicki et al., 2013). Another neuropeptide implicated in regulating body bends and bending angles is NLP-49. NLP-49 is released from the AVK interneuron and functions through the receptor SEB-3. Mutants in *nlp-49* show reduced mid-body and hip bends, while NLP-49 overexpression leads to hyperactive locomotion (Chew et al., 2018a).

**FIGURE 3:**
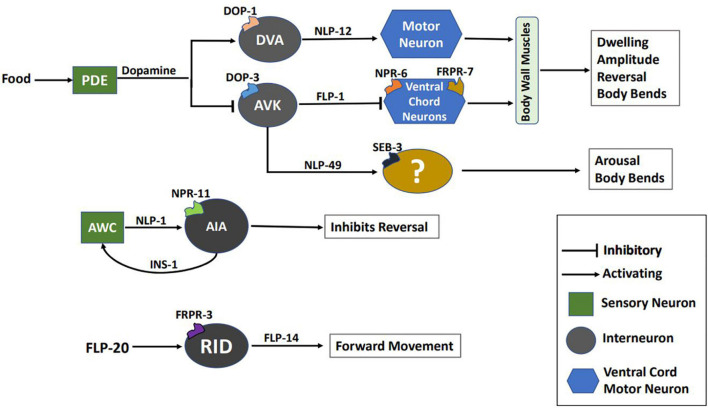
Schematic of multiple neuropeptidergic circuits underlying locomotion. This image has been partially adapted from Oranth et al. (2018).

In addition to the parameters discussed above, other aspects of locomotion in *C. elegans*, including reversals, Ω turns, and speed are also modulated by neuropeptides. Reversals and Ω turns are essential for reorientation during foraging, mate search, and aversion. On the other hand, speed determines locomotion rate during aversion, arousal (a state of hyperactive locomotion), and sleep. Studies have implicated the FLP-18 neuropeptide in the control of reversal frequency and the reversal length (Cohen et al., 2009; Bhardwaj et al., 2018). FLP-18 functions through its receptors’ NPR-1, NPR-4, and NPR-5. In *flp-18* mutants, reversal frequency decreases, and the reversal length increases during the local search (Cohen et al., 2009; Bhardwaj et al., 2018, 2020). This implies that *flp-18* mutants fail to perform local search effectively. Interestingly, the reversal frequency in *flp-18* mutants does not change significantly during the transition from local to global search (Cohen et al., 2009). As a result, global search is impaired in these animals. Various other neuropeptides modulate the dynamics of exploration. NLP-1 and INS-1 control the magnitude of reversals, where NLP-1 is released from the AWC sensory neurons, which also secretes glutamate. Both NLP-1 and glutamate bind to their receptors NPR-11 and GLC-3, respectively, on the AIA interneuron. Glutamate/GLC-3 is an inhibitory synapse that promotes reversals, while NLP-1/NPR-11 reduces reversal frequency. INS-1, released from AIA neurons, modulates the activity of AWC neurons and functions in the same process as NLP-1 (Chalasani et al., 2010). Exploration is also modulated by genes encoding pigment dispersing factor signaling components, including PDF-1, PDF-2, and their receptor PDFR-1. Mutants in *pdf-1, pdf-2*, or *pdfr-1* fail to explore larger areas and show reduced speed (Flavell et al., 2013). Mutants in the galanin-like receptor *npr-9* show decreased reversals, and Ω turns (Campbell et al., 2016). Previous studies in mice have shown that perfusion of the Galanin-like peptide (GALP) reduces locomotor activity in mice (Kauffman et al., 2005). Another neuropeptide, FLP-20 ensures the *C. elegans* stay on food by decreasing reversal frequency. *flp-20* mutants show an increased reversal rate in off food conditions, suggesting its role in promoting dispersal (Rabinowitch et al., 2016). Delving further into the functioning of FLP-20, it has been reported to modulate speed during arousal in response to a mechanosensory stimuli. FLP-20 functions through FRPR-3, which acts in the RID interneuron (Chew et al., 2018b). The RID neuron, being a specialized neuroendocrine cell, releases the neuropeptide FLP-14, required for maintaining forward movement. Mutants in *flp-14* have impaired forward movement and exhibit frequent pauses and increased reversal frequency (Lim et al., 2016). The function of some of these neuropeptides is illustrated in Figure 3. FLP-2 (functional analog of mammalian orexin) and PDF-1 have also been reported to modulate locomotion during arousal where FLP-2 functions through the receptor FRPR-18, a functional analog of mammalian orexin type-2 receptor (Chen et al., 2016). Behaviors like developmentally timed quiescence and sleep require reduced locomotor activity. These behaviors require the function of multiple neuropeptides, including FLP-11, FLP-24, FLP-13, NLP-8, and NLP-22 that regulate stopping of locomotion during quiescence and sleep (Nelson et al., 2013, 2014; Nath et al., 2016; Steuer Costa et al., 2019). Mating in *C. elegans* requires turning back or reversing during copulation and inhibition of reorientations during mate search. PDF-1 is required to inhibit reorientation in males during mate search to explore large areas (Janssen et al., 2009; Barrios et al., 2012). Neuropeptides including FLP-8, FLP-10, FLP-12, and FLP-20 regulate turning during mating (Liu et al., 2007).

In addition to solid substrates, *C. elegans* also inhabit liquid media and use swimming as their mode of locomotion in liquid. Several neuropeptides have been implicated in regulating the swimming rate of *C. elegans.* The swimming rate is quantified as the number of body bends per unit time. Mutants in *flp-18, flp-3, flp-10*, and *flp-21* show increased swimming rates, while *flp-9* mutants show the opposite phenotype of lower swimming rate (Chang et al., 2015).

Apart from *C. elegans*, neuropeptides regulate locomotion in arthropods, mollusks, and vertebrate systems (Henry et al., 2000; Kahsai et al., 2010b; Li et al., 2015). Some of these neuropeptidergic signaling pathways are conserved across phyla. In *Drosophila melanogaster*, the neuropeptide, *Drosophila* tachykinin (DTK) provides spatial orientation during exploration while another neuropeptide, short neuropeptide F (sNPF), fine-tunes locomotion and regulates speed of the animal (Kahsai et al., 2010a). In migratory locusts, *Locusta migratoria*, two related neuropeptides NPF1a and NPF2, regulate locomotion during the transition of the locust from solitary to the swarming phase (Hou et al., 2017). In mice, neuropeptide S functions through the corticotropin releasing factor receptor 1 (CRF1) to increase locomotory activity (Li et al., 2015). CRF1 shows similarity in structure and function with the SEB-3 GPCR in *C. elegans* (Jee et al., 2013). Several mood-related disorders like anxiety and depression are attributed to dysregulation of CRF signaling [reviewed in Arborelius et al. (1999)]. Another group of neuropeptides that are opiodergic including enkephalin and dynorphin have been shown to control locomotion and dyskinesia in parkinsonian rat models (Sgroi et al., 2016).

The discussed observations imply that neuropeptides play a vital role during locomotion and dysregulation in neuropeptidergic signaling could result in severe locomotory defects. The presence of some conserved signaling pathways allows researchers to extrapolate these circuits to vertebrates and humans to start to unravel the complex wiring of locomotion circuitry involving neuropeptides.

### Feeding Behavior

Feeding is an indispensable process for survival, influencing a wide range of behavioral repertoire by an organism. Despite its simple structure, *C. elegans* exhibits a variety of physiological and behavioral changes in response to food availability and nutritional status. For instance, behaviors like foraging, mating, egg-laying, dauer formation, quiescence, social interactions, etc., are affected by the feeding state of the animal [reviewed in Avery and You (2012), Barrios (2014)]. *Caenorhabditis elegans* is a bacterivorous worm, largely maintained on a slow-growing strain of *E. coli* bacteria, i.e., OP50 strain under standard laboratory conditions. It ingests the bacterial food through pumping and peristaltic movements of the pharynx [reviewed in Avery and You (2012)]. The feeding process and related behaviors are highly regulated by neuromodulators released by the somatic and pharyngeal nervous systems. These comprise of many neuropeptides and biogenic amines such as serotonin, octopamine, tyramine, etc., which control feeding and locomotion in a food-dependent manner (Horvitz et al., 1982; Rex et al., 2004; Alkema et al., 2005; Suo et al., 2006). The study of neuropeptide processing mutants, i.e., *egl-3* and *egl-21*, paved the way to understand the importance of neuropeptides in feeding and/or fat storage [(Husson et al., 2006, 2007) and reviewed in Holden-Dye and Walker (2013); Srinivasan (2020)]. Further, the significance of neuropeptides is established by the neuropeptide release mutants, *unc-31* that exhibit constitutive pharyngeal pumping during starvation, suggesting an underlying reduction in neuromodulation (Avery et al., 1993; Dalliere et al., 2016).

**FIGURE 4:**
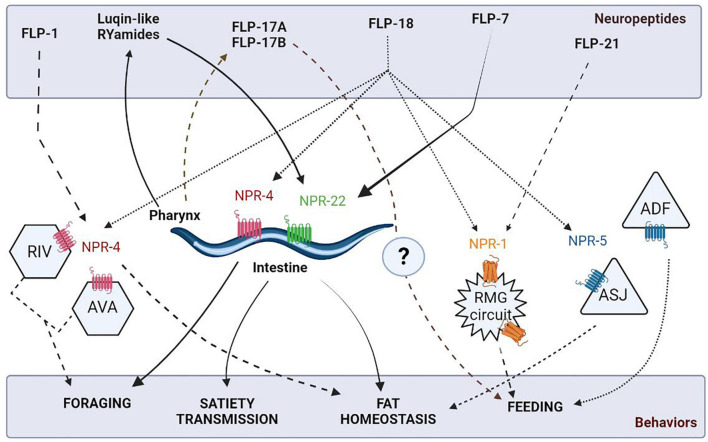
Schematic of neuropeptidergic circuits underlying feeding related behaviors. The figure has been adapted from Cohen et al. (2009).

Literature has reported the role of diverse neuropeptides such as FLPs, ILPs, and NLPs in the feeding circuit. The widely expressed family of FLP neuropeptides act on GPCR receptors to modulate feeding behavior in *C. elegans*. For instance, FLP-1 is implicated in fat storage and diet-induced changes in antioxidant responses mediated *via* the NPR-4 receptors in the intestine (Mutlu et al., 2020; Jia and Sieburth, 2021). Also, *npr-4* mutants result in impaired foraging behavior, fat homeostasis, and food preference (Cohen et al., 2009; Yu et al., 2016; Bhardwaj et al., 2018). Another allatostatin/galanin-like GPCR, viz, NPR-9 impinges on the AIB interneurons to regulate food-evoked foraging, which, in turn, may affect metabolism and fat storage (Bendena et al., 2008). Interestingly, the FLP-18 neuropeptide has been found to act on different GPCRs to regulate a variety of feeding-related functions, i.e., FLP-18 binds to NPR-4 receptors to regulate fat accumulation in the intestine and modulate foraging behavior in the RIV and AVA neurons (Cohen et al., 2009). Moreover, NPR-5 receptors mediate the effects of FLP-18 in the ciliated sensory neurons to induce lipid storage in ASJ neurons to regulate dauer formation and in ADF neurons to stimulate feeding (Cohen et al., 2009; Lemieux et al., 2015). Multiple features of feeding behaviors are illustrated in Figure 4. Further, FLP-18 and FLP-21 are implicated in the social feeding behavior, by associating with the different isoforms of NPR-1 receptors (de Bono and Bargmann, 1998; Rogers et al., 2003). It is noteworthy that the NPR-1 receptor is an evolutionary conserved Neuropeptide Y (NPY) receptor, known for its role in regulating feeding in vertebrates [reviewed in Beck (2006)]. NPY and its receptor defects have been shown to contribute to eating disorders such as anorexia nervosa and obesity [reviewed in Gehlert (1999), Zhang et al. (2012)].

More recent studies have shown that a tachykinin-like peptide, FLP-7, secreted by the ASI neurons has been found to act on the intestinal NPR-22 receptors to promote fat mobilization (Palamiuc et al., 2017). The pharyngeal circuit in *C. elegans* also secretes FLP-17A and FLP-17B neuropeptides, indicating their possible role in promoting feeding (Dalliere et al., 2016). A group of highly conserved insulin-like neuropeptides has been shown to determine the on and off states during pharyngeal pumping in *C. elegans*. These include INS-1 peptide, which acts by increasing the 5-HT sensitivity and insulin signaling pathway mutants, i.e., *daf-2* and *daf-18* mutants involved in promoting feeding adaptation (Dwyer and Aamodt, 2013; Dillon et al., 2016). Insulin-based regulation of feeding is extremely important as its malfunctioning could result in several metabolic disorders such as obesity, heart disease, and diabetes [reviewed in Kolb et al. (2020)]. Intriguingly and showing similarities to vertebrate systems, *C. elegans* possess endogenous opioids like NLP-24 that act through the opioid receptor, NPR-17, to modulate feeding by stimulating pharyngeal pumping during starvation in worms (Cheong et al., 2015). Moreover, feeding states have been found to influence decision-making between attractive and aversive stimuli along with foraging *via* PDF-2/PDFR-1 dependent neuropeptidergic signaling (Ghosh et al., 2016; O’Donnell et al., 2018). In parallel, the Luqin-like RYamide peptides secreted from the pharyngeal M1 and M2 neurons, induce food-evoked satiety as a negative feedback loop. These peptides primarily exert their actions *via* the NPR-22 receptors on the feeding pacemaker MC and serotonergic RIH neurons (Ohno et al., 2017).

The identification of highly conserved cholecystokinin (CCK)-gastrin-like peptides, viz, DYRPLQFamide (NLP-12a) and DGYRPLQFamide (NLP-12b) in *C. elegans* has helped to illuminate the pathways of satiety transmission and fat storage in mammals. Janssen et al. report that the *nlp-12* and *ckr-2* receptor mutants show an increased fat accumulation, indicating a mechanism operating by alleviated metabolism of fat stores (Janssen et al., 2008). The functional conservation of CCKs is also complemented by another study, where expression of the mammalian CCK-8 degrading enzyme, tripeptidyl peptidase II (TPPII) in *C. elegans* fat cells resulted in decreased fat accumulation (McKay et al., 2007).

Despite noteworthy strides in functionally characterizing the roles of neuropeptides in regulating feeding behavior, their numerous non-cell-autonomous endocrine effects are yet to be understood. Future investigations in this area could aid in addressing prevailing metabolic disorders such as obesity, diabetes, etc.

### Mating Behavior

**FIGURE 5:**
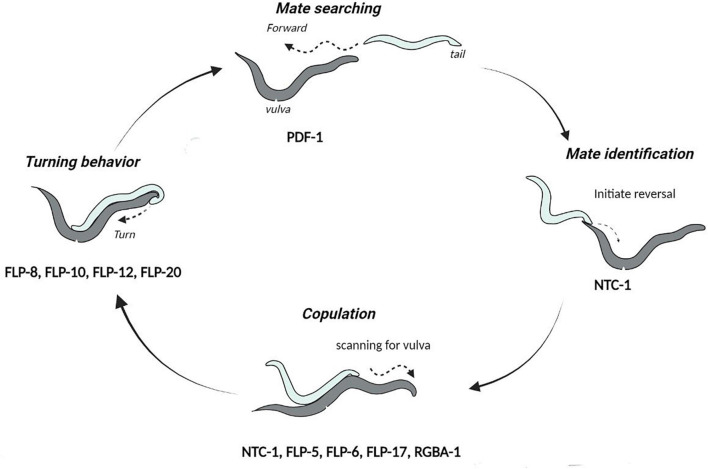
Cycle of events during mating in *C. elegans.* The neuropeptides involved in each step of the mating behaviors are indicated below the step they function at. This image has been adapted from Sherlekar et al. (2013).

*Caenorhabditis elegans* mating is a complex behavior, comprising of the coordinated execution of spatio-temporal motor actions. In an androdioecious species like *C. elegans*, males initiate and execute the mating process while hermaphrodites essentially play a passive role [reviewed in Sherlekar and Lints (2014)]. Of the total 385 neurons present in the *C. elegans* male, at least 79 are known to facilitate mating (Liu and Sternberg, 1995; Molina-Garcia et al., 2020). The stereotyped mating begins due to the influence of chemical pheromones, i.e., ascarosides which attract the male to contact and scan its mate for detecting the vulva [reviewed in Chute and Srinivasan (2014)]. Once the vulva is located, the male stops scanning and inserts its protracted copulatory spicules inside the hermaphrodite animal to transfer sperm [reviewed in Barr and Garcia (2006)]. Thus, the mating process involves five different steps: mate identification, reversals, repetitive turning, vulva detection, and finally intromission (illustrated in Figure 5). A plethora of literature indicates the role of neurotransmitters like dopamine, serotonin, octopamine, etc., in regulating mating behaviors in *C. elegans*. While dopamine is involved in the mating motivation, sperm transfer, and recovery from post-coital lethargy, serotonin influences the ventral-tail curling during male turning (Loer and Kenyon, 1993; Carnell et al., 2005; Correa et al., 2012; LeBoeuf et al., 2014). On the other hand, octopamine, the biological equivalent of norepinephrine in invertebrates, acts downstream of dopamine to regulate the locomotor activity state in copulating mates (Suo et al., 2019).

Apart from neurotransmitters, neuropeptides are known to play a pivotal role in modulating the mating circuit. Here, we review the neuropeptides implicated in mating behaviors of *C. elegans*. The alleged role of neuropeptides in this behavior was initially observed by examining the neuropeptide processing *egl-3* mutants, defective in egg-laying and other mechanosensory responses that could be important during mating (Kass et al., 2001). The commencing step of the mating process, i.e., mate-searching behavior is regulated by the PDF-1 (Pigment-dispersing factor) neuropeptide released by the AIM neuron, which promotes the expression of DAF-7/TGFβ in the male-specific ASJ neurons. PDF-1 modulation circuit also extends to URY, PQR, and PHA neurons due to PDFR-1 receptor expression in these neurons (Barrios et al., 2012; Hilbert and Kim, 2018). Interestingly, PDF-1 also displays phylogenetic conservation in other organisms, for instance, regulation of male sex drive in *Drosophila melanogaster* (Fujii and Amrein, 2010). Further, defects in the orthologs of PDFR-1 receptors in humans are known to result in anhedonia-related disorders such as bipolar disorder and post-traumatic stress disorders (Soria et al., 2010; Ressler et al., 2011).

The function of neuropeptides in the mating process is further established by the neuropeptide-release *unc-31* mutants, required for the release of Dense-core vesicles (DCVs). These mutants are unable to initiate spicule insertion and hence, fail to transfer sperms into the vulva. However, the other steps of mating behavior remain unaffected in *unc-31* mutants (LeBoeuf and Garcia, 2017). In addition, the coherence of all these mating steps is governed by the oxytocin/vasopressin-like peptide known as nematocin. Nematocin (NTC-1) is released by the male ray DVA neuron and acts on the NTR-1 receptor to promote response to the potential mates and integrate different steps of the mating behavior (Garrison et al., 2012). In most species studied, these oxytocin-related neuropeptides display genetic and functional conservation in reproduction-related behaviors, such as selection of mate, copulation and offspring care (Wagenaar et al., 2010; Melis and Argiolas, 2011; Garrison et al., 2012; Marlin et al., 2015). Apart from oxytocin/vasopressin like peptides, the FLP neuropeptides including FLP-5, FLP-6, and FLP-17 are suggested to modulate the spicule circuit upon stimulation of the male RnA ray neurons (Lints et al., 2004). Liu et al. (2007), have additionally shown that FLP-8, FLP-10, FLP-12, and FLP-20 neuropeptides participate in sensory transduction during male sexual turning behaviors. In parallel, a FLP neuropeptide orthologs in *Drosophila*, i.e., neuropeptide F and the mammalian neuropeptide Y are known to influence male courtship (Lee et al., 2006). More recent work has shown that glia-derived neuropeptides in *C. elegans* potentially explain the age-related decline in male mating behaviors, RGBA-1 neuropeptides acting on the NPR-28 receptor in serotonergic and dopaminergic neurons influence mating efficiency. Noteworthy is that the polymorphic alleles of the *rgba-1* gene are associated with male virility and were found to alleviate mating behavior deterioration in aging worms (Yin et al., 2017). The fact that diverse neuropeptides are involved in the multiple overlapping steps of mating behaviors, highlights the intricate ways by which they modulate the mating circuit. However, it is unclear how these neuropeptides differentially participate in the anticipatory phase, i.e., arousal in response to pheromones and instinctive drive, or the consummatory phase, i.e., execution of the mating process.

### Sleep-Like Behavior

**FIGURE 6:**
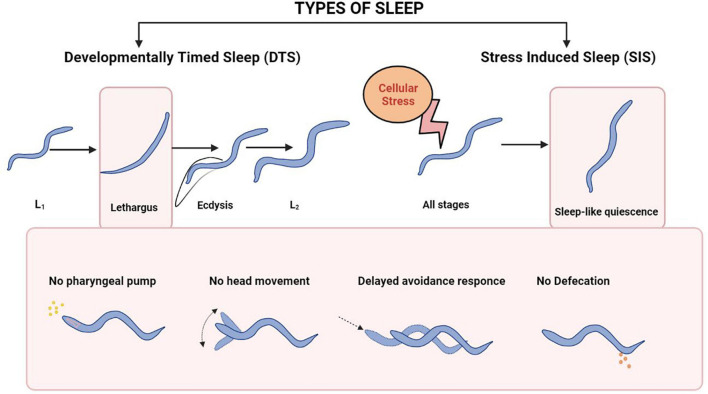
Sleep patterns in *C. elegans.* The top panel shows the types of Sleep in *C. elegans*. The bottom Panel shows the physiological events during each type of sleep. The bottom panel has been adapted from Nath et al. (2016).

Molecular mechanisms underlying sleep have been studied using multiple model organisms including invertebrate models like *Drosophila melanogaster*, and *Danio rerio* (Hendricks et al., 2000; Shaw et al., 2000; Zhdanova et al., 2001; Mackiewicz et al., 2008). A quiescent state known as lethargus before each molt during development is referred to as developmentally timed sleep (DTS, illustrated in Figure 6). *Caenorhabditis elegans* also shows a state of sleep-like quiescent behavior during which there is a temporary halt in locomotion, pharyngeal pumping, head movement, defecation and feeding [(Cassada and Russell, 1975; Nath et al., 2016) and illustrated in Figure 6].

**FIGURE 7:**
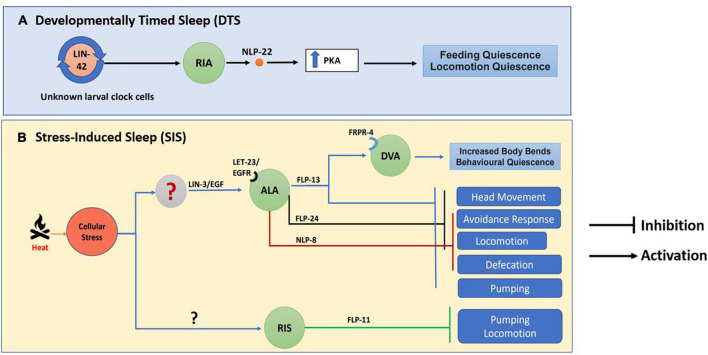
Neuropeptide circuitry regulating sleep. The image indicates **(A)** Developmentally Timed Sleep (DTS) and **(B)** Stress Induced Sleep (SIS) along with the neuropeptides involved in each process. This image has been adapted from work by Nelson et al. (2013, 2014) and Nath et al. (2016).

*Caenorhabditis elegans* has been used as a model for studying lethargus behaviors induced by a variety of genes. For instance, cyclic guanosine monophosphate (cGMP) dependent protein kinase (EGL-4) boosts sleep-like state in *C. elegans* (Raizen et al., 2008). Neuropeptides play an important role in the sleep/wake cycle of vertebrates [reviewed in Sutcliffe and de Lecea (2002)]. However, mechanistic insights into neuropeptide function in sleep-like behavior comes in large part from work on *D. melanogaster* and *C. elegans*. In *Drosophila* the Pigment-Dispersing Factor (PDF) neuropeptide is responsible for normal circadian rhythm (Renn et al., 1999). *Caenorhabditis elegans* also secrete PDF-1 from the RMG neuronal circuit and the secretion of PDF-1 is lowered during lethargus (Choi et al., 2013). Neuropeptide NLP-22, structurally similar to the mammalian Neuromedin S peptide (NMS), released from the RIA interneuron allows for sleep-like behavior and functions through protein kinase A (PKA) dependent mechanisms (Nelson et al., 2013) and illustrated in Figure 7A.

Apart from DTS, cellular stress like heat, cold, tissue damage, and hypertonicity also results in a stress-induced quiescence state or stress-Induced sleep (SIS) in *C elegans*, [(Hill et al., 2014) and illustrated in Figure 4]. Again, neuropeptides play a crucial role in SIS-like behavior. The neuropeptide receptor NPR-1 modulates heat stress-induced sleep-like behavior during hyperoxic conditions (Soto et al., 2019). Heat stress causes the release of LIN-3/EGF, which acts on its receptor LET-23/EGFR present on the ALA neuron, triggering the release of FLP-13 neuropeptide that is required for feeding and locomotory quiescent behavior (Nelson et al., 2014). The FLP-13 neuropeptide released from the ALA neuron as a result of stress acts on the receptor FRPR-4 on the DVA neuron to modulate the posture of the animals during quiescence (Nelson et al., 2015). The transcription factor LIM-6 controls both peptidergic and GABAergic function in the RIS neuron. LIM-6 maintains the expression of the APTF-1 transcription factor, which in turn upregulates the expression of FLP-11 neuropeptide required for sleep behavior in RIS neurons (Turek et al., 2016). Studies have also shown that triple mutants in the neuropeptides *nlp-8*, *flp-24*, and *flp-13* synergistically inhibit SIS-like behavior (Nath et al., 2016). This experiment shows that different neuropeptidergic signaling mechanisms could contribute collectively toward sleep behavior (illustrated in Figure 7B).

Neuropeptidergic control of sleep is conserved in higher organisms as well. In zebrafish multiple neuropeptides maturing from proprotein RFamide neuropeptide VF (NPVF) act synergistically to promote sleep (Lee et al., 2017). NPY has been shown to modulate sleep patterns in rats and also affects sleep endocrine systems in patients facing depression (Held et al., 2006; Szentirmai and Krueger, 2006). Neuropeptide-S, an evolutionary conserved neuropeptide regulates sleep-wake cycle in mammals (Xu et al., 2004). Another neuropeptide hypocretin (also known as orexin) promotes wakefulness and inhibits sleep in mammals [reviewed in Soya and Sakurai (2020)]. Narcolepsy, a neurological condition affecting sleep has been associates with dysregulation of orexin signaling. The discovery of these neuropeptidergic pathways in invertebrates and vertebrate models, can answer many questions related to molecular mechanisms underlying sleep and hence may be of importance in the field of neuropsychiatric disorders related to sleep.

### Learning and Memory

Learning and memory are crucial biological properties for an organism to survive in its habitat. Multiple invertebrate models are used for understanding the mechanisms behind memory and learning (Carew and Sahley, 1986). For instance, *C. elegans* and *D. melanogaster* allow for intricate genetic manipulations of the nervous system, which in turn can provide insight into molecular mechanisms involved in the process of memory formation and cognitive functions (Cerutti and Levin, 2006).

*Caenorhabditis elegans* typically shows two types of learning, associative learning and non-associative learning that induce different degrees of memory based on the training paradigms used (Wen et al., 1997; Morrison et al., 1999). Different paradigms could result in Long-Term Memory (LTM), Short-Term Memory (STM), and Intermediate-Term Memory (ITM) (Rose et al., 2002, 2003; Steidl et al., 2003; Li et al., 2013; Dahiya et al., 2019). Another important learning paradigm involves understanding the interactions between *C. elegans* with their surrounding microbes. For instance, *C. elegans* can detect and discriminate infectious microbes like *Pseudomonas aeruginosa*, through its innate immune system [reviewed in Nicholas and Hodgkin (2004)].

Pathogen avoidance learning is known to be dependent on signaling through insulin-like peptides (ILPs), such as INS-11, secreted by the intestinal cells (Lee and Mylonakis, 2017). During this process ILPs act through different sets of neurons for sensing external signals, for instance, INS-16 through the pheromone-sensing neuron ADL and INS-4 through the bacteria-sensing neuron AWA (Wu et al., 2019). Further, FLP-20 has been reported to be required for STM in the mechanosensory neurons after mass training (Li et al., 2013). FLP-20 neuropeptides from primary mechanosensory neurons bind to their receptor FRPR -3 which is present on the neuroendocrine cell RID, thus controlling arousal behavior in *C. elegans* (Chew et al., 2018b). Further, the Neuropeptide-Like Protein-38 (NLP-38)/Myo Inhibitory Peptide (MIP) signal activates the G protein-coupled receptor SPRR-2 which is responsible for salt aversive learning (Peymen et al., 2019). The evolutionarily conserved Neuromeric U (NMU) neuropeptide family homolog CAPA-1 in *C. elegans*, is secreted from ASG neurons and alongwith its receptor NMUR-1 is required for the retrieval of learned salt avoidance behavior (Watteyne et al., 2020). In order to promote learning, ILPs have been shown to play antagonistic roles. Chen *et al.*, have reported that INS-6 from ASI neurons suppresses the expression of INS-7 in URX neurons to enable learning (Chen et al., 2013). Similarly, FLP-34, released from the serotonergic neurons, acts through the NPR-11 receptor on the AIA interneuron for negative associative learning (Fadda et al., 2020).

Interestingly, the administration of one such neuropeptide, i.e., Neuropeptide S (NPS) into the APP/PSI mouse model of Alzheimer’s disease (AD), has been shown to result in the reduction of β-Amyloid plaques indicating the clinical relevance of employing neuropeptides in treating age related disorders of the brain (Zhao et al., 2019). Neuropeptide S is also shown to promote olfactory, and spatial memory in rodent models (Wang et al., 2020). These studies bring out the importance of understanding the role of neuropeptides in different forms of learning and memory.

### Addiction Behavior

Substance abuse is a growing concern of societies around the world. It refers to the illicit and/or excessive use of psychoactive drugs, including alcohol. Chronic use of these drugs alters the expression of several key players in the neuronal substrate resulting in a state of tolerance and gradual addiction. Apart from voluntary priming to these drugs, stress has been attributed to one of the leading causes of addiction [reviewed in Schank et al. (2012)]. Alcohol is a commonly used drug, and the physiological effects associated with it have been studied extensively. Alcohol induces effects in a dose-dependent manner ranging from dysregulation in limb coordination, impaired speech at lower doses to even death at higher doses. Although several genes are implicated in addiction behavior, the mechanism by which binge episodes of alcohol consumption lead to addiction, however, remain largely elusive. *Caenorhabditis elegans* shows sedation and defects in locomotion in response to alcohol at a concentration similar to that seen in humans (Davies et al., 2003; Mitchell et al., 2007; Pandey et al., 2021). Therefore, *C. elegans*, with a simple nervous system and easy genetic manipulation, serves as a good model system for studying addiction behaviors and their underlying molecular mechanism. *Caenorhabditis elegans* exhibit multiple behaviors in response to attractant or aversive cues. Chemotaxis and locomotion changes are some of the well-established behaviors that can be utilized for addiction studies. In this part of the review, we will be focusing on the role of neuropeptides in addiction.

Most studies regarding addiction focus on circuitry that controls tolerance, withdrawal, and relapse pathways. Dopamine signaling, for that matter, has been well studied [reviewed in Wise (2004), Hyman et al. (2006)]. Another important gene encoding the calcium sensitive potassium (BK) channel has gained attention as one of the key players in addiction behavior. BK channels are activated to mediate behavioral responses to alcohol with mechanisms that may be conserved across multiple systems (Davies et al., 2003; Pietrzykowski and Treistman, 2008; Kreifeldt et al., 2013; Velazquez-Marrero et al., 2016).

The role of neuropeptides in addiction related behaviors is poorly understood. However, hints indicating possible roles for neuropeptides in addictive behaviors in vertebrate systems and *C. elegans* have started to emerge (Mitchell et al., 2010; Schank et al., 2012). An intriguing study by Thiele *et al.*, has opened a new avenue indicating the role of neuropeptide signaling in addiction. They show that neuropeptide Y (NPY) levels inversely control the ethanol intake and resistance in rats. The study further reports that animals lacking NPY are resistant to ethanol induced effects even though the plasma concentration of ethanol is similar to controls showing the behavior (Thiele et al., 1998). Infusion of NPY in CNS also shows reduced intake of alcohol post tolerance in rats (Thorsell et al., 2007). Previous evidence has indicated that NPY levels are higher in ethanol preferring rats than in ethanol non-preferring animals (Ehlers et al., 1998). This neuroadaptive circuit functioning through NPY is conserved, and in *C. elegans* acts through NPY receptor-like protein, NPR-1. Ethanol exposure leads to downregulation of the NPR-1 pathway suggesting that the development of acute tolerance is negatively regulated by NPR-1 signaling (Davies et al., 2004).

Addiction-related withdrawal and relapse are mainly associated with a negative emotional state of elevated anxiety and stress and CRF, being a stress neuropeptide, has been shown to mediate these behaviors [reviewed in Heilig and Koob (2007), Koob (2008)]. The levels of CFR increase during the ethanol withdrawal period and subsequently decrease after ethanol intake (Merlo Pich et al., 1995; Olive et al., 2002). The withdrawal related anxiogenic effect is relieved in animals treated with CRF antagonist alarmin or alpha-helical CRF confirming the role of CFR in withdrawal associated behavior (Rassnick et al., 1993; Funk and Koob, 2007). As discussed till now, it is clear that CFR and NPY exert opposing effects in ethanol related behaviors. The mechanism for this antagonism was parsed out recently (Pleil et al., 2015). These exciting results show that NPY activation inhibits CFR neurons through a G_*i*_-mediated PKA-dependent postsynaptic mechanism to reduce withdrawal response and ethanol intake. Likewise, in *C. elegans*, a CFR receptor-like protein, SEB-3, positively regulates acute tolerance to ethanol. SEB-3 is a potential receptor for neuropeptide NLP-49 and mutants in *seb-3 (gf)* phenocopy withdrawal behavior showing increased tremors in *C. elegans* (Jee et al., 2013). Endogenous cannabinoid signaling has also been parsed out in *C. elegans*. A putative neuropeptide receptor NPR-19 (mammalian homolog of CB_1_ receptor) mediates the cannabinoid signaling in these animals and inhibits the aversive response to nociception (Oakes et al., 2019).

The puzzle is still far from complete, and many more pieces are yet to be found. Although neuropeptide research regarding addiction has recently gained momentum, a plethora of questions remains unanswered. Interesting to note here is that several candidate ligands of neuropeptide receptors, already implicated in addiction, are potential candidates for screening. Moreover, neuropeptides released in response to stress and regulating arousal can be interesting molecules for further studies. Even though some receptors are known to play an essential role in ethanol-induced behavior, their site of action and their circuitry are still largely unknown. Together, these findings and further work with multiple model organisms can serve an important role in designing potential therapeutics to treat relapse and reward behaviors associated with substance abuse.

## Conclusion

Organisms bring change in their activities in response to both intrinsic and extrinsic cues. These changes are referred to as the organism’s behavior. In this review, we have summarized the results from various studies to understand the complex mechanisms underlying behaviors and how neuropeptides regulate them. We also provide insight into multiple neuropeptide-based behaviors using *C. elegans* as a model system. Neuropeptides, once released, are not re-uptaken and therefore continue transmitting information until they are degraded or their signal is inhibited [reviewed in Russo (2017)]. Hence, neuropeptides are interesting molecules to study sustained physiological responses. In this regard, neuropeptides are emerging as crucial modulators of several behaviors, including some discussed in this review.

As discussed in this review, neuropeptidergic signaling plays a pivotal role in relaying the information between the neurons during the execution of behaviors and is conserved across the phyla. For instance, Neuropeptide Y is one of the major neuromodulators of feeding, sleep, memory, and learning in humans. Likewise, in *C. elegans*, Neuropeptide Y/RFamide- like receptors called NPR regulates a broad spectrum of behaviors, including feeding, locomotion, mating, etc., Neuropeptide or neuropeptide receptor mutants show behavioral defects which can be easily scored as a phenotype for analyses. These behavioral studies have helped in developing several *C. elegans* disease models to extrapolate the findings to human diseases. *Caenorhabditis elegans* based disease models for multiple conditions including epilepsy, autism and neurodegenerative disorders continue to allow is to understand the molecular pathways and biomarkers associated with these diseases (Dexter et al., 2012; Wong et al., 2018; Rawsthorne et al., 2021).

With emerging researchers’ interest in investigating the role of neuropeptides in relation to behavioral studies, a plethora of questions remain unanswered. (1) How multiple neuropeptides act in concert to execute a particular behavior? (2) Identifying the pleiotropic effects of a neuropeptide through its multiple sites of action. (3) Deciphering the crosstalk between different neuropeptides during complex behaviors. (4) Understanding stimulus-response relationship due to slow and extended-release of neuropeptides.

Addressing these and other questions will help to elucidate the complex mechanism of behaviors and could allow for developing therapeutic strategies to treat the disorders associated with defects in neuropeptide signaling.

## Author Contributions

UB, NS, and SS researched and wrote the manuscript. KB supervised and helped editing the manuscript. All authors contributed to the article and approved the submitted version.

## Conflict of Interest

The authors declare that the research was conducted in the absence of any commercial or financial relationships that could be construed as a potential conflict of interest.

## Publisher’s Note

All claims expressed in this article are solely those of the authors and do not necessarily represent those of their affiliated organizations, or those of the publisher, the editors and the reviewers. Any product that may be evaluated in this article, or claim that may be made by its manufacturer, is not guaranteed or endorsed by the publisher.
